# Surveillance of the Incidence and Mortality of Oral and Pharyngeal, Esophageal, and Lung Cancer in Nevada: Potential Implications of the Nevada Indoor Clean Air Act

**DOI:** 10.3390/ijerph18157966

**Published:** 2021-07-28

**Authors:** Kevin Foote, David Foote, Karl Kingsley

**Affiliations:** 1 Department of Clinical Sciences, University of Nevada, Las Vegas, NV 89106, USA; footek1@unlv.nevada.edu (K.F.); footed1@unlv.nevada.edu (D.F.); 2 Department of Biomedical Sciences, University of Nevada, Las Vegas, NV 89106, USA

**Keywords:** oral cancer, epidemiology, incidence, mortality, Nevada

## Abstract

Reviews of national and state-specific cancer registries have revealed differences in rates of oral, esophageal, and lung cancer incidence and mortality that have implications for public health research and policy. Many significant associations between these types of cancers and major risk factors, such as cigarette usage, may be influenced by public health policy such as smoking restrictions and bans—including the Nevada Clean Indoor Air Act (NCIAA) of 2006 (and subsequent modification in 2011). Although evaluation of general and regional advances in public policy have been previously evaluated, no recent studies have focused specifically on the changes to the epidemiology of oral and pharyngeal, esophageal, and lung cancer incidence and mortality in Nevada. Methods: Cancer incidence and mortality rate data were obtained from the National Cancer Institute (NCI) Division of Cancer Control and Population Sciences (DCCPS) Surveillance, Epidemiology and End Results (SEER) program. Most recently available rate changes in cancer incidence and mortality for Nevada included the years 2012–2016 and are age-adjusted to the year 2000 standard US population. This analysis revealed that the overall rates of incidence and mortality from these types of cancer in Nevada differs from that observed in the overall US population. For example, although the incidence rate of oral cancer is decreasing in the US overall (0.9%), it is stable in Nevada (0.0%). However, the incidence and mortality rates from esophageal cancer are also decreasing in the US (−1.1%, −1.2%, respectively), and are declining more rapidly in Nevada (−1.5%, −1.9%, respectively). Similarly, the incidence and mortality rates from lung are cancer are declining in the US (−2.5%, −2.4%, respectively) and are also declining more rapidly in Nevada (−3.2%, −3.1%, respectively). Analysis of previous epidemiologic data from Nevada (1999–2003) revealed the highest annual percent change (APC) in oral cancer incidence in the US was observed in Nevada (+4.6%), which corresponded with the highest APC in oral cancer mortality (+4.6%). Subsequent studies regarding reduced rates of cigarette use due to smoking restrictions and bans have suggested that follow up studies may reveal changes in the incidence and mortality rates of oral and other related cancers. This study analysis revealed that oral cancer incidence rates are no longer increasing in Nevada and that mortality rates have started to decline, although not as rapidly as the overall national rates. However, rapid decreases in both the incidence and mortality from esophageal and lung cancer were observed in Nevada, which strongly suggest the corresponding changes in oral cancer may be part of a larger epidemiologic shift resulting from improved public health policies that include indoor smoking restrictions and bans.

## 1. Introduction

Reviews of national and state-specific cancer registries have revealed many differences in oral and pharyngeal cancer incidence and mortality that have profound implications for public health research and policy [1,2]. For example, some studies have revealed divergent trends in oral cancer incidence based on regional differences in socioeconomic demographics [3]. Other research has confirmed these associations and has provided more specific details evaluating shared risk factors, which may contribute to these regional differences [4].

Regional and state-level analyses have revealed many significant associations between oral and pharyngeal cancers with major risk factors, such as cigarette usage, that may also be influenced by public health policy such as smoking restrictions and bans [5,6]. For example, recent changes to smoke-free workplace policies and other smoking bans have resulted in significant changes in cigarette usage, as well as secondhand smoke exposures [7,8]. Studies of the results of these changes were used as justification in 2011 to modify and expand the Nevada Clean Indoor Air Act (NCIAA), which was originally passed in 2006 to reduce primary usage of cigarettes and tobacco products in public places and to reduce secondhand smoke exposure [9,10].

The NCIAA was designed to protect both children and adults from cigarette smoke and second-hand cigarette smoke in the majority of public spaces and places of employment [11]. The overall goal of this legislation was to reduce the availability of, and provide disincentives towards, cigarette smoking and to reduce the environmental effects of smoking-related aerosols including second-hand smoke, from public and private school buildings, childcare facilities, grocery and drug stores, indoor restaurants and shopping or retail establishments, video arcades, movie theaters and government buildings—although some casino gaming areas were exempted [12]. Modification of these protocols was made to include vaping and e-cigarettes in 2011, with evaluations of air quality and particulate matter from casino non-smoking areas demonstrating significant reductions in smoking related aerosols and particulate matter (PM2.5) compared with smoking areas, *R* = 0.71, *p* = 0.005 [13], which may lead to improved public health and reductions in smoking-related cancers.

Although evaluation of general and regional advances in public policies around smoking have been evaluated, no recent studies have focused specifically on the evaluation of the effects of these changes to related cancer incidence and mortality in Nevada, such as oral, esophageal and lung cancers. To date, only two studies (both published more than ten years ago) have evaluated these trends in oral cancers in Nevada, which may have changed significantly in the intervening decade [14,15]. Based upon the lack of recent analysis and epidemiologic data, the primary objective of this study was to evaluate the recent changes to the epidemiology of both rates of incidence of, and mortality from these smoking-related cancers in Nevada.

## 2. Materials and Methods

### 2.1. Source Data and Analysis

Cancer incidence and mortality data for this study were obtained from the National Cancer Institute (NCI) Division of Cancer Control and Population Sciences (DCCPS) Surveillance, Epidemiology and End Results (SEER) program [16]. This program collects incidence and survival data from approximately 34.6% of the United States (US) population designed for public use and dissemination [17]. These data are compiled through the National Center for Health Statistics (NCHS), which evaluated and compiled data regarding cancers of the oral cavity and pharynx.

The US and Nevada oral and pharyngeal, esophageal, and lung cancer incidence and mortality statistics in this study were generated using the SEER database program (NCHS, Hyattsville, MD, USA) (SEER*Stat). Methods used to determine rates and trends have been previously reported [15]. In brief, these data are obtained from the National Vital Statistics System public use data files and are compiled and analyzed by the NCHS using the Join point Trend Analysis statistical software. This program evaluates and models the natural logarithm of any selected incidence or mortality rate, provides identification of the years during which any observed changes in trend are noted, and provides summary statistics or graphics, based upon this analysis.

### 2.2. Statistical Analysis of Incidence and Mortality Trends

The most recent rate changes in cancer incidence and mortality in Nevada available included the years 2002–2017, age-adjusted to the year 2000 standard US population. This included data regarding specific cancer sites, including esophagus, lung and bronchus, and oral cavity (and pharynx) cancers. The changes in overall trends over time were calculated and graphed based on data from 2002–2017, dividing this most recent updated data (2008–2017) by earlier rates (e.g., 2000–2008). The percentage change in rates was graphed in box and whisker plots along with the 95% confidence intervals, which were provided by the Join point Trend Analysis for each cancer site, sub-group, and geographic location.

## 3. Results

**Figure 1:**
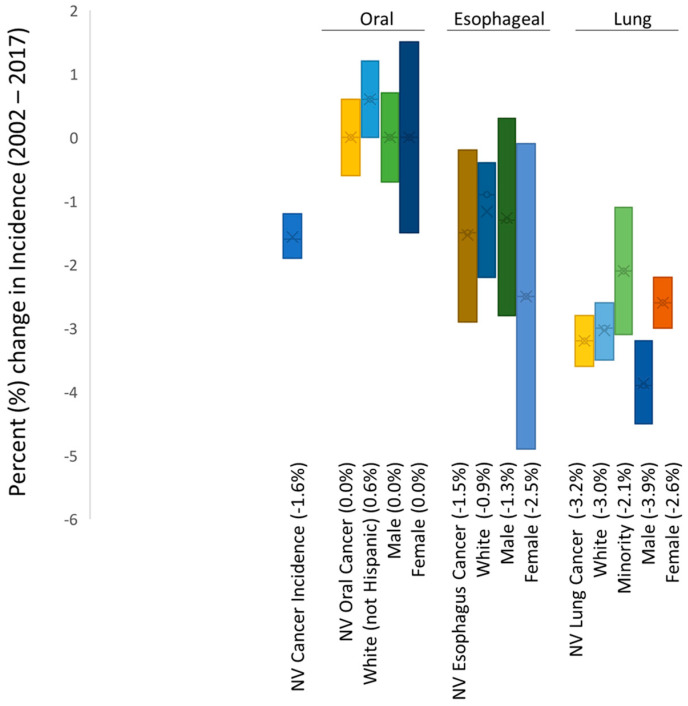
Analysis of smoking-related cancer incidence rates in Nevada 2002–2017. Differential results demonstrated stable rates of oral cancer incidence (0.0%) with declining rates of esophageal cancer incidence (−1.5%) associated mainly with females (−2.5%), as well as declining rates of lung cancer incidence (−3.2%), which were most strongly associated with declining rates among males (−3.9%). Note: insufficient data were available to analyze or plot non-White oral and esophageal cancer incidence in Nevada.

Analysis of the epidemiologic data from Nevada revealed an overall decrease in the rates of cancer incidence (all cancers) of −1.6% (Figure 1). More detailed analysis of these data stratified by cancer type revealed oral cancer incidence rates in Nevada as stable (0.0% change) with a slight increase observed among White (non-Hispanics) of 0.6% and no changes observed among males or females (0.0%, 0.0%, respectively). However, further analysis of these data also revealed reductions in the rates of esophageal cancer in Nevada over this same time period (2013−2017) of −1.5%, which appeared to be most strongly associated with reductions among females (−2.5%). In addition, reductions were also observed among lung cancer incidence rates (−3.2%), which appeared to be most strongly associated with robust reductions among males (−3.9%).

An analysis of the overall (national) incidence of these cancers (oral and pharyngeal, esophageal, lung and bronchus) was compiled to determine if these trends in incidence within Nevada followed broader national epidemiologic trends (Table 1). These data demonstrated that trends in the incidence for all cancers were similar between the US and Nevada (−1.0%, −1.6%, respectively). In addition, the trends in the incidence of oral cancer were also similar between Nevada and the US overall (0.0%, −0.9%, respectively). Moreover, the incidence trends among esophageal cancers (−1.5%, −1.1%) and lung cancers (−3.2%, −2.0%) also demonstrated similar trends between Nevada and the US overall. It should be noted that insufficient data were available to analyze non-White (minority) oral and esophageal cancer incidence in Nevada.

**Figure 2:**
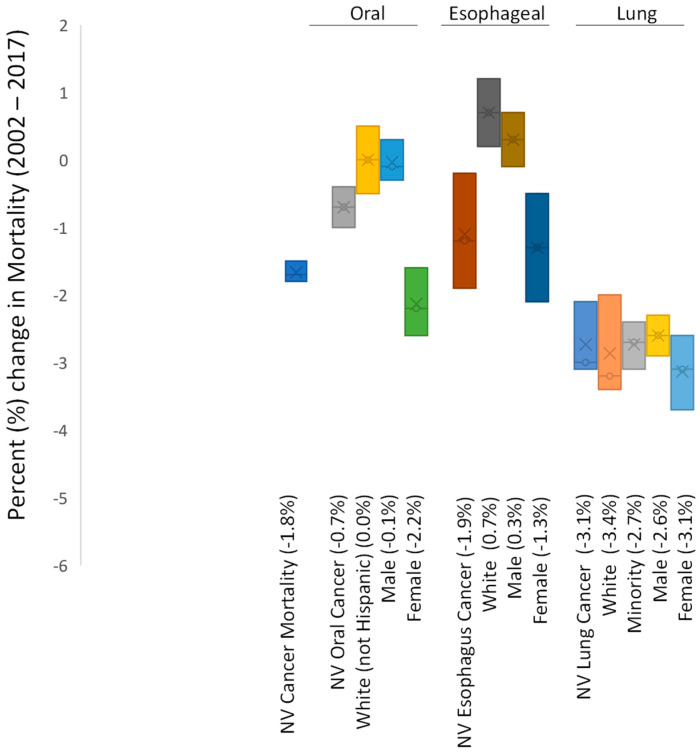
Analysis of smoking-related cancer mortality rates in Nevada 2002−2017. Differential results revealed declining rates of oral cancer mortality (−0.7%) strongly associated with females (−2.2%), as well as declining rates of esophageal cancer mortality (−1.9%) also associated mainly with females (−1.3%). Declining rates of lung cancer mortality (−3.1%) were observed with similar rates among males and females (−2.6%, −3.1%, respectively). Note: insufficient data were available to analyze or plot non-White oral and esophageal cancer mortality in Nevada.

Analysis of the epidemiologic data from Nevada revealed an overall decrease in the rates of cancer mortality (all cancers) of −1.8% (Figure 2). More detailed analysis of these data stratified by cancer type revealed oral cancer mortality rates in Nevada as declining (−0.7%) with the majority of these decreases observed among females (−2.2%). In addition, these data also revealed more significant reductions in the mortality rates of esophageal cancer in Nevada over this same time period (−1.9%), which also appeared to be most strongly associated with reductions among females (−1.3%). Finally, reductions were also observed among lung cancer mortality rates (−3.1%), which appeared to be similar among males and females (−2.6%, −3.1%, respectively).

Finally, an analysis of the overall (national) mortality of these cancers (oral, esophageal, lung) was compiled to determine if these trends in mortality within Nevada are similar with national epidemiologic trends (Table 2). These data demonstrated that trends in the mortality for all cancers were similar between the US and Nevada (−1.5%, −1.8%, respectively). In addition, the trends in the mortality from oral cancer were lower Nevada than the US overall (−0.7%, 0.5%, respectively). In addition, the mortality trends in Nevada compared with the US for esophageal cancers (−1.9%, −1.2%) and lung cancers (−3.1%, −2.4%) also demonstrated similar trends. Similar to the incidence data, it was noted that insufficient data were available to analyze non-White (minority) oral and esophageal cancer mortality in Nevada.

## 4. Discussion

The primary objective of this study was to elucidate and evaluate the recent changes to the epidemiology of both incidence and mortality from oral, as well as esophageal and lung cancers in Nevada. The results of this current study revealed that oral cancer incidence and mortality in Nevada is similar with the US overall, a noteworthy development since the last time these data were analyzed and published [15]. The previous study in 2008 found that oral cancer incidence and mortality were increasing rapidly in Nevada (approximately 4.6%) and were the highest in the US—therefore, the results of this current study demonstrate a dramatic shift to an oral cancer incidence that is now stable (at approximately 0.0%) with oral cancer mortality declining (−0.7%), which are similar to the national trends. Similar but more robust declines in the incidence and mortality from esophageal and lung cancer were also observed in Nevada.

However, the changes in incidence were not equally distributed among all demographic groups—a finding similar to the previous report from nearly a decade ago [14]. The current study found that although rates of esophageal and lung cancer incidence in Nevada are decreasing, they are decreasing more rapidly among females in Nevada—supporting other epidemiologic studies suggesting males nationwide, and especially those in Nevada may exhibit lowered responses to health prompts and higher resistance to tobacco cessation efforts and their resulting effects on cancer incidence [18,19]. Similarly, the rates of oral and esophageal cancer mortality are also decreasing in Nevada, mainly among females—which further demonstrates the strong links observed between cancer incidence and mortality [1,5,6]. However, the lack of available data regarding incidence and mortality among minorities is concerning, given that previous observations have revealed that non-White (minority) populations may be more vulnerable to, and more likely to suffer from health disparities in general, and more specifically from smoking-related cancers—such as oral and pharyngeal, esophageal, and lung cancers [20,21].

The results of this study are significant, as no recent available evidence has evaluated any changes to related smoking-related cancer incidence and mortality in Nevada, including oral, esophageal, and lung cancers. The original epidemiologic study from this group in 2008 outlined the increased trend in both oral cancer incidence and mortality in Nevada, which were the highest in the US at that time [14]. The subsequent follow-up study analyzing behavioral risk factors such as smoking following the legislative clean air act (NCIAA) suggested that decreased smoking might subsequently change cancer incidence and mortality significantly in the intervening decade [15]. This study provides the first observations of these data and provide detailed analysis of population subgroups, such as racial and ethnic minorities that are often at greater risk for health disparities and poor cancer outcomes [22]. These data confirm and support other studies that have demonstrated changes to behavioral risk factors, such as decreased smoking prevalence, may be linked with decreases in cancer incidence and subsequent decreases in cancer mortality [23,24,25].

Although these data are significant, there are other behavioral and epidemiologic risk factors besides cigarette use and smoking that contribute to the development, incidence and mortality of these cancers, such as the influence of alcohol consumption [26]. Although most epidemiologic studies attribute the majority of oral, esophageal and lung cancer risk to cigarette and tobacco usage, some significant portion of risk may also be attributable to other high-risk behavioral patterns, including heavy alcohol use [27]. Because oral cancer risk may be related significantly, in part, to alcohol consumption and Nevada may be associated with high rates of alcoholism and heavy drinking—these data and the analysis of the potential effects of the NCIAA may only partially explain the patterns in cancer trends observed in this study [28].

## 5. Conclusions

Previous epidemiologic studies from Nevada found the highest oral cancer incidence and mortality in the US was observed in Nevada, although subsequent studies following the NCIAA observed reduced rates of cigarette use following these smoking restrictions and bans—suggesting that positive changes might subsequently be observed in the incidence and mortality rates of oral and other related cancers. This study analysis revealed that oral cancer incidence rates are no longer increasing in Nevada and that mortality rates have started to decline, although not as rapidly as the overall national rates. However, rapid decreases in both the incidence and mortality from esophageal and lung cancer were observed in Nevada, which strongly suggest the corresponding changes in oral cancer may be part of a larger epidemiologic shift resulting from improved public health policies that included the NCIAA indoor smoking restrictions and bans.

## Figures and Tables

**Table 1:** Comparison of National (US) and Nevada-specific incidence rates of oral and pharyngeal, esophageal, and lung cancers.

	Nevada	United States (US)
Incidence (all cancer) (95% Confidence Intervals)	−1.6% (−1.9, −1.2)	−1.0% (−1.2, −0.8)
Oral Cancer-Incidence	0.0% (−0.6, 0.6)	−0.9% (−3.8, 2.1)
Race—White (not Hispanic)	0.6% (0.0. 1.2)	−0.6% (−3.7, 2.5)
Race—Black (plus Hispanic)	* Insufficient data	−1.7% (−1.9, −1.4)
Sex—Male	0.0% (−0.7, 0.7)	0.8% (0.6, 1.0)
Sex—Female	0.0% (−1.5, 1.5)	0.4% (0.2, 0.5)
Esophagus Cancer-Incidence	−1.5% (−2.9, −0.2)	−1.1% (−1.4, −0.9)
Race—White (not Hispanic)	−0.9% (−2.2, −0.4)	−0.5% (−0.8, −0.2)
Race—Black (plus Hispanic)	* Insufficient data	−4.3% (−4.5, −4.1)
Sex—Male	−1.3% (−2.8, −0.3)	−0.8% (−1.5, −0.2)
Sex—Female	−2.5% (−4.9, −0.1)	−1.3% (−1.5, −1.1)
Lung Cancer-Incidence	−3.2% (−3.6, −2.8)	−2.0% (−2.2, −1.9)
Race—White (not Hispanic)	−3.0% (−3.5, −2.6)	−1.8% (−2.0, −1.6)
Race—Black (plus Hispanic)	−2.1% (−3.1, −1.1)	−2.6% (−3.0, −2.3)
Sex—Male	−3.9% (−4.5, −3.2)	−2.8% (−2.9, −2.6)
Sex—Female	−2.6% (−3.0, −2.2)	−1.3% (−1.5, −1.1)

* Note: Due to differing state reporting requirements some demographic data may be missing resulting in “insufficient data”.

**Table 2:** Comparison of National (US) and Nevada-specific mortality rates of oral and pharyngeal, esophageal and lung cancers.

	Nevada	United States (US)
Mortality (all cancer) (95% Confidence Intervals)	−1.8% (−1.9, −1.6)	−1.5% (−1.6, −1.5)
Oral Cancer-Mortality	−0.7% (−1.0, −0.4)	0.5% (0.1, 1.0)
Race—White (not Hispanic)	0.0% (−0.0. 0.5)	0.7% (0.3, 1.0)
Race—Black (plus Hispanic)	* Insufficient data	−0.3% (−1.0, 0.4)
Sex—Male	−0.1% (−0.3, 0.3)	0.4% (0.1, 0.8)
Sex—Female	−2.2% (−2.6, −1.6)	−3.4% (−9.0, 2.6)
Esophagus Cancer-Mortality	−1.9% (−2.1, −0.2)	−1.2% (−1.3, −1.0)
Race—White (not Hispanic)	0.7% (0.2, 1.2)	−2.2% (−4.3, −0.1)
Race—Black (plus Hispanic)	* Insufficient data	−4.6% (−4.7, −4.4)
Sex—Male	0.3% (−0.1, 0.7)	−1.1% (−1.3, −0.9)
Sex—Female	−1.3% (−2.1, −0.5)	−1.5% (−1.7, −1.4)
Lung Cancer-Mortality	−3.1% (−3.6, −2.8)	−2.4% (−2.6, −2.3)
Race—White (not Hispanic)	−3.2% (−3.4, −2.8)	−2.5% (−2.7, −2.2)
Race—Black (plus Hispanic)	−2.7% (−3.1, −2.4)	−2.6% (−2.9, −2.4)
Sex—Male	−2.6% (−2.9, −2.3)	−3.1% (−3.2, −2.9)
Sex—Female	−3.1% (−3.7, −2.6)	−1.8% (−2.0, −1.6)

* Note: Due to differing state reporting requirements some demographic data may be missing resulting in “insufficient data”.

## Data Availability

The data presented in this study are available publicly on the Surveillance, Epidemiology, and End Results Program (SEER) website and database (https://seer.cancer.gov).

## Abbreviations

Nevada Clean Indoor Air Act (NCIAA), National Cancer Institute (NCI), Division of Cancer Control and Population Sciences (DCCPS), Surveillance, Epidemiology and End Results (SEER), United States (US).
